# Supplementary material for: Detecting Interspecific Positive Selection Using Convolutional Neural Networks
Source: Mol Biol Evol. 2025 Jun 30;42(7):msaf154. doi: 10.1093/molbev/msaf154 (PMC12287699; doi:10.1093/molbev/msaf154)
Supplement: msaf154_Supplementary_Data [file msaf154_supplementary_data.pdf]

# Detecting interspecific positive selection using convolutional neural networks — Supplementary Information

**Charlotte West**[*,1], **Conor R. Walker**[*,1,2,a], **Shayesteh Arasti**[1,b], **Viacheslav Vasilev**[1,c], **Xingze Xu**[1,d], **Nicola De Maio**[1], **and Nick Goldman**[1,**]

[*]These authors contributed equally to this work.

[1]European Molecular Biology Laboratory, European Bioinformatics Institute (EMBL-EBI), Wellcome Genome Campus, Hinxton, CB10 1SD, United Kingdom

[2]Department of Genetics, University of Cambridge, Cambridge, CB2 3EH, United Kingdom

[a]Current address: conor@conorwalker.net

[b]Current address: Computer Science and Engineering Department, University of California San Diego, La Jolla, CA 92093, USA

[c]Current address: Moscow Institute of Physics and Technology, Phystech School of Applied Mathematics and Computer Science, Dolgoprudny, 141701 Russia

[d]Current address: Cambridge Stem Cell Institute, Cambridge, CB2 0AW, UK

[**]Corresponding author: E-mail: goldman@ebi.ac.uk.

# 6 Supplementary Information

## 6.1 Roadmap to creating and using CNNs for detecting positive selection

Our code, available at https://github.com/charlotte-west/OmegaAI, is not a ready-to-use tool for all scenarios but provides the scripts used to simulate data, train, and evaluate OmegaAI models. Users may find it useful for developing their own models for positive selection detection. In this section, we outline a roadmap for users seeking to investigate the presence or absence of positive selection in the evolution of a set of homologous, protein-coding gene sequences from different species using the methods described in this work. This approach can be extended to multi-gene or genome-wide scans for positive selection, provided the underlying gene tree and evolutionary rates are consistent or highly similar. This roadmap is intended to be read alongside the main publication text and the code on GitHub.

### 6.1.1 Pre-analysis

The pre-analysis for this roadmap briefly describes common, existing protocols for the preparation and execution of detecting selection for multi-species alignments and phylogenetic analysis in general. We therefore encourage readers to explore each aspect of this pre-analysis more deeply for enhanced understanding of the steps, which may have more optimal solutions for their particular problem (see Anisimova et al., 2013; Yang, 2014; Zou et al., 2024, for a non-exhaustive list of possible further reading).

To begin, identify your gene of interest. Next, retrieve the nucleotide sequences of homologous genes from different species. Our findings suggest that sequence divergence plays a crucial role in detecting positive selection. Specifically, homologous genes with branch lengths around 0.2 substitutions per codon per branch yielded the most reliable results. This level of divergence provides a strong selection signal when present while minimising information loss due to high divergence (see also Álvarez-Carretero, Kapli, and Yang, 2023). On the other hand, OmegaAI results were strong compared to our benchmark likelihood methods for high divergences, making our OmegaAI a potentially favourable strategy when dealing with highly-divergent sequences.

Next, align the sequences using a high-quality aligner such as PRANK (Löytynoja and Goldman, 2008), then infer the phylogenetic tree representing their evolutionary relationships. These steps can be performed manually by performing a BLAST search (Camacho et al., 2009) on the gene of

interest, collecting homologs, aligning them with PRANK, and using the resulting multiple sequence alignment (MSA) to infer a tree with software such as IQ-TREE (Minh et al., 2020). Alternatively, precomputed gene trees are available in Ensembl for many genes, offering a convenient alternative. Various methods exist for constructing gene trees from a single gene; see Anisimova et al. (2013) and Zou et al. (2024) for further details.

### 6.1.2 Simulating training data

The next step involves simulating training data for training and testing a CNN. By using simulated sequences, the ground truth regarding positive selection is known, allowing the CNN to learn from labelled data. OmegaAI performs best when applied to datasets very similar to the ones it was trained on. We have shown this to be the case for the divergence scaling of the tree and is likely the case for the tree topology too.

To generate training data, simulate sequence evolution along the phylogenetic tree constructed in the pre-analysis stage, which represents the evolutionary relationships among your homologous gene sequences. We used INDELible (Fletcher and Yang, 2009) for this, though other software options exist that can achieve similar results (see Ly-Trong et al., 2022, for alternatives). The key requirement of the sequence evolution model is that it allows variation in selective pressure, including the possibility of positive selection. In our work, we employed an extension of the GY94 model that includes indels, with the parameter $\omega$ used to distinguish between sequence evolution with and without positive selection.

We found that training OmegaAI with 1,000,000 simulated datasets produced a highly effective binary classifier when benchmarked against likelihood-based methods. While 1,000,000 may be more than strictly necessary, simulations are computationally inexpensive and do not present a bottleneck. To prevent class imbalance, we simulate equal proportions of data with and without positive selection. Details on parameter selection can be found in the Methods section.

Simulated sequences should be aligned before training. A fast but less accurate aligner, such as Clustal (Sievers and Higgins, 2018) or MAFFT (Katoh and Standley, 2013), seems adequate or even preferable. This choice is motivated by two factors: first, OmegaAI performed similarly or better when trained on MSAs with more alignment errors, likely due to increased exposure to real-world alignment inaccuracy; second, highly accurate alignment does not significantly improve training outcomes but introduces unnecessary computational overhead.

Lastly, we recommend maintaining consistent alignment row ordering so that the CNN can learn how best to use the relative levels of relatedness without having an explicit phylogenetic tree input (see section 3.5 in the main paper). To maximise this feature, we recommend taxon ordering reflect evolutionary relatedness, for example, ordering taxa in the MSA as they would appear in a Newick file.

#### 6.1.3 Training

OmegaAI models are convolutional neural networks (CNNs) implemented in Python using TensorFlow (Abadi et al., 2016). Their architecture, described in section 5.3, is similar to that of Suvorov, Hochuli, and Schrider (2020). Users can adopt a similar architecture and expect comparable performance when applying their model to similar data. A detailed description of the architecture and software is provided in the Methods section. Although various machine learning methods—such as logistic regression, random forests, recurrent neural networks, and transformers—can perform categorical classification (binary, in our case), we did not explore these alternatives in this study (see Mukhamediev et al., 2022, for a review of machine learning technologies).

Users may wish to conduct their own tests to determine an appropriate stopping point or implement an automated stopping criterion, determining the number of epochs to train for which balances overfitting and underfitting (see Terry, Jayakumar, and De Alwis, 2021, for examples of stopping criteria). CNN training is computationally intensive and benefits from parallel processing. We recommend using a GPU with at least 8 GB of memory to enable efficient parallel processing and reduced training time compared to CPU-only setups. Exact memory requirements will depend on the size of the dataset and model complexity.

#### 6.1.4 Testing

Once your model has been trained, and before analysing your own data, we recommend generating a simulated test dataset of approximately 2,000 sequences. This allows the user to benchmark their model's performance and select an appropriate classification threshold. Specifically, we suggest evaluating this test set using the trained CNN (and optionally a likelihood-based method such as CODEML) and plotting ROC and PR curves. These analyses help determine a classification threshold suited to your needs—for example, prioritising precision over recall—and ideally selecting a threshold that achieves more true positives at the same or lower FPR than the likelihood-based

method, with 5% being a commonly accepted FPR in likelihood frameworks.

While OmegaAI models benefited from training on less-accurate alignments, our results indicate that alignment accuracy is a critical factor in the accurate detection of positive selection. To maximise model performance, we suggest using the same high-accuracy aligner for both your real and test data, to get the best estimate of how your CNN model is likely to perform on your data.

Finally, analyse your gene MSA using your CNN and obtain a binary classification indicating whether or not this the gene has evolved under positive selection as inferred by the model. This can be repeated and scaled up for many genes or a genome-wide scan.

## References


Abadi, M. et al. (2016). TensorFlow: large-scale machine learning on heterogeneous distributed systems. arXiv:1603.04467.

Álvarez-Carretero, S., P. Kapli, and Z. Yang (2023). Beginner's guide on the use of PAML to detect positive selection. Molecular Biology and Evolution 40, msad041.

Anisimova, M. et al. (2013). State-of the art methodologies dictate new standards for phylogenetic analysis. BMC evolutionary biology 13, 1–8.

Camacho, C. et al. (2009). BLAST+: architecture and applications. BMC bioinformatics 10, 1–9.

Fletcher, W. and Z. Yang (2009). INDELible: a flexible simulator of biological sequence evolution. Molecular Biology and Evolution 26, 1879–1888.

Katoh, K. and D. M. Standley (2013). MAFFT multiple sequence alignment software version 7: improvements in performance and usability. Molecular Biology and Evolution 30, 772–780.

Löytynoja, A. and N. Goldman (2008). Phylogeny-aware gap placement prevents errors in sequence alignment and evolutionary analysis. Science 320, 1632–1635.

Minh, B. Q. et al. (2020). IQ-TREE 2: new models and efficient methods for phylogenetic inference in the genomic era. Molecular biology and evolution 37, 1530–1534.

Mukhamediev, R. I. et al. (2022). Review of artificial intelligence and machine learning technologies: Classification, restrictions, opportunities and challenges. Mathematics 10, 2552.

Sievers, F. and D. G. Higgins (2018). Clustal Omega for making accurate alignments of many protein sequences. Protein Science 27, 135–145.

Suvorov, A., J. Hochuli, and D. R. Schrider (2020). Accurate inference of tree topologies from multiple sequence alignments using deep learning. Systematic Biology 69, 221–233.

Terry, J. K., M. Jayakumar, and K. De Alwis (2021). Statistically significant stopping of neural network training. arXiv:2103.01205.

Ly-Trong, N., S. Naser-Khdour, R. Lanfear, and B. Q. Minh (2022). AliSim: a fast and versatile phylogenetic sequence simulator for the genomic era. Molecular Biology and Evolution 39, msac092.

Yang, Z. (2014). Molecular Evolution: a Statistical Approach. Oxford, UK: Oxford University Press.

Zou, Y. et al. (2024). Common methods for phylogenetic tree construction and their implementation in R. Bioengineering 11, 480.

### 6.2 Supplementary Figures

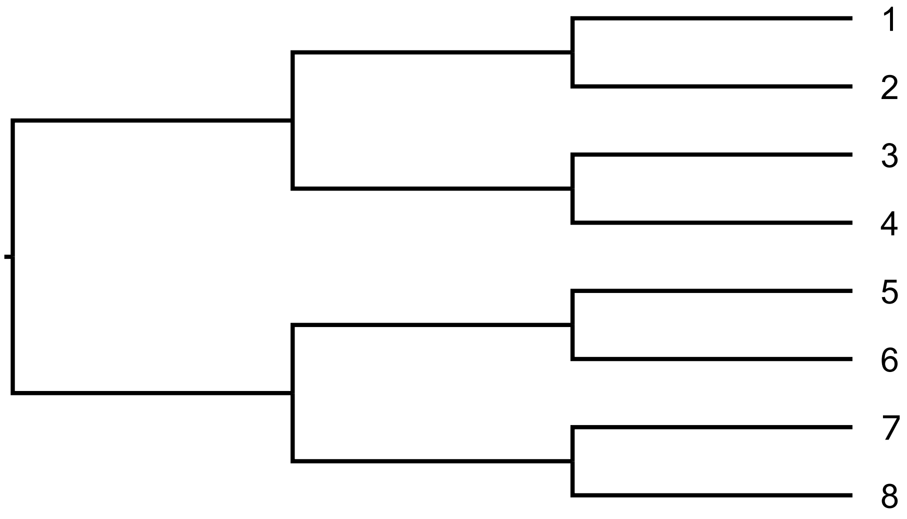


**Figure S 1. Artificial phylogenetic tree used for simulation.** An 8-taxon, symmetric, ultrametric tree with equal branch lengths scaled to a chosen divergence. Simulated sequences are evolved along this tree using INDELible (Fletcher and Yang, 2009) and associated MSAs have consistent row ordering relative to the ordering of the tips shown in the tree. For the baseline parameter set, each branch length is set to 0.2 expected substitutions per codon, making the root to tip length of the tree 0.6.

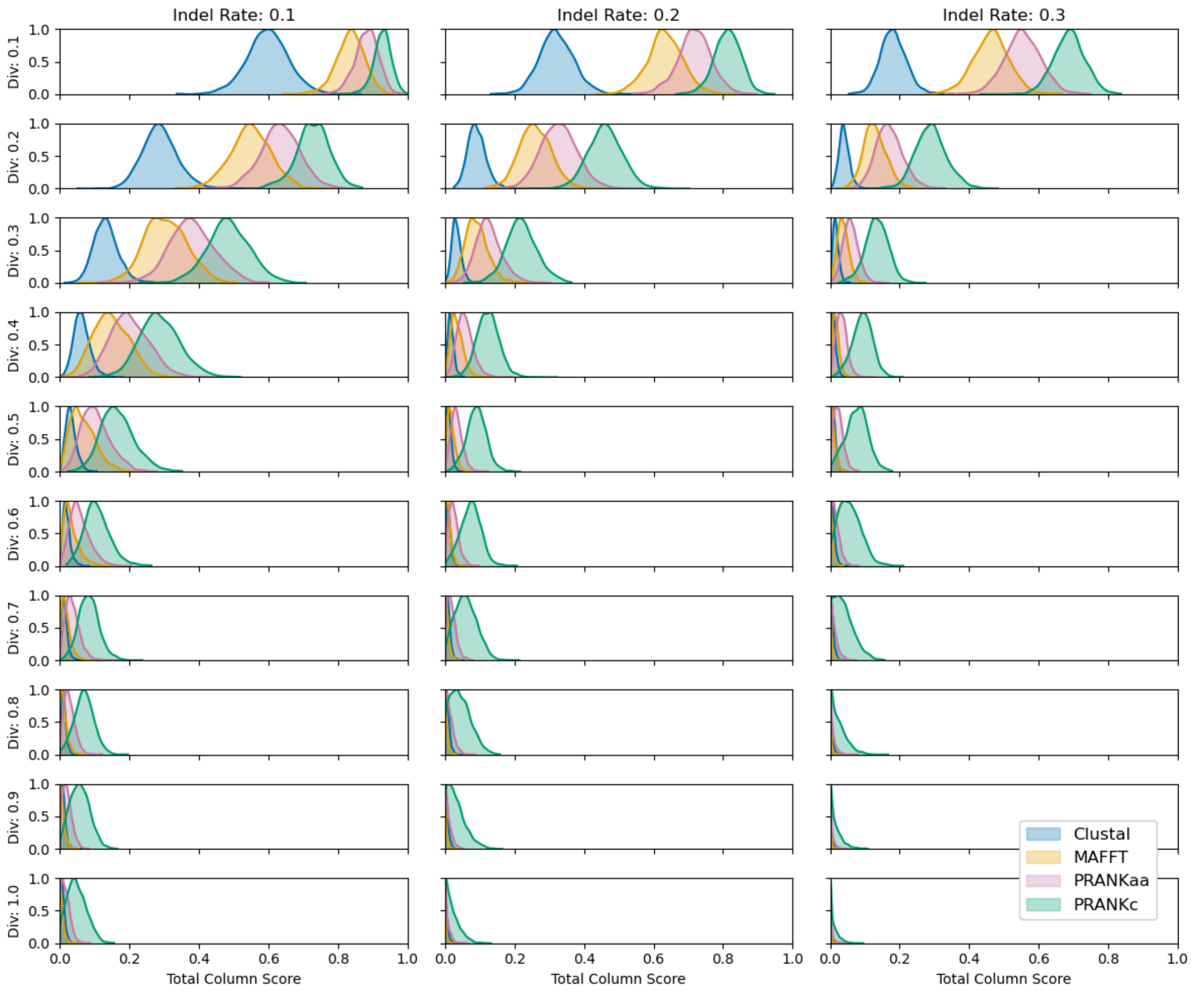


**Figure S 2. Alignment Total Column Scores across aligners, divergences and indels.** For each divergence-indel combination 2,000 sets of sequences along with their true MSAs were generated using INDELible (see Methods). For each set of sequences the alignment is then inferred using Clustal, MAFFT, and two PRANK versions (PRANKaa and PRANKc), forming the test sets in this study. The Total Column Score (proportion of true alignment columns in the inferred alignment) was calculated for each alignment using FastSP (Mirarab and Warnow, 2011) to assess alignment quality, which decreases as divergence and indel rates increase.

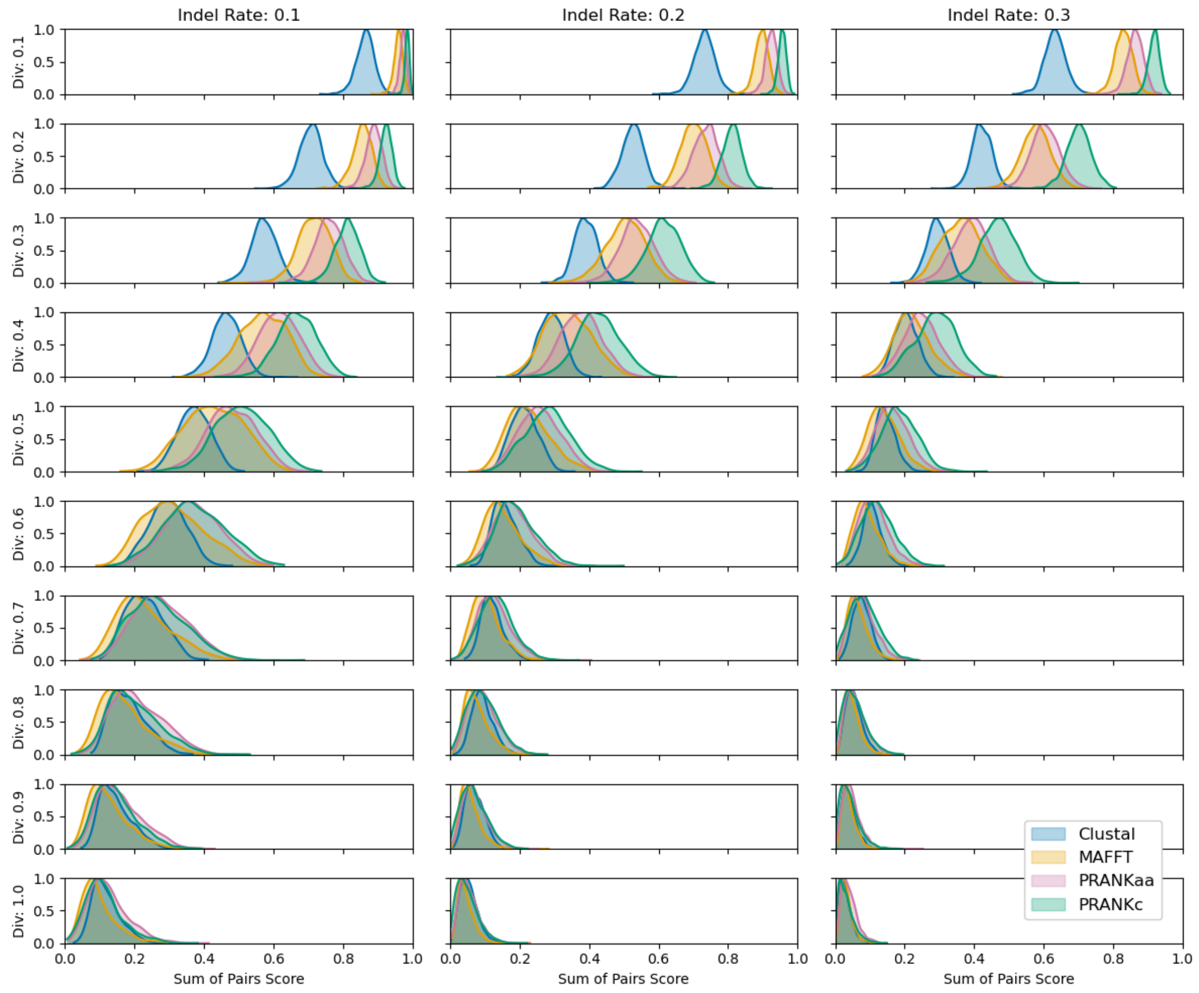


**Figure S 3. Alignment Sum of Pairs Scores across aligners, divergences and indels.** Details are as in Supplementary Fig. 2, except that here we show the Sum of Pairs Score, which is the proportion of true homologies (aligned pairs) found in an inferred alignment. The Sum of Pairs Score (here calculated with FastSP: Mirarab and Warnow, 2011), another measure of the quality of an alignment, decreases as divergence and indel rates increase.

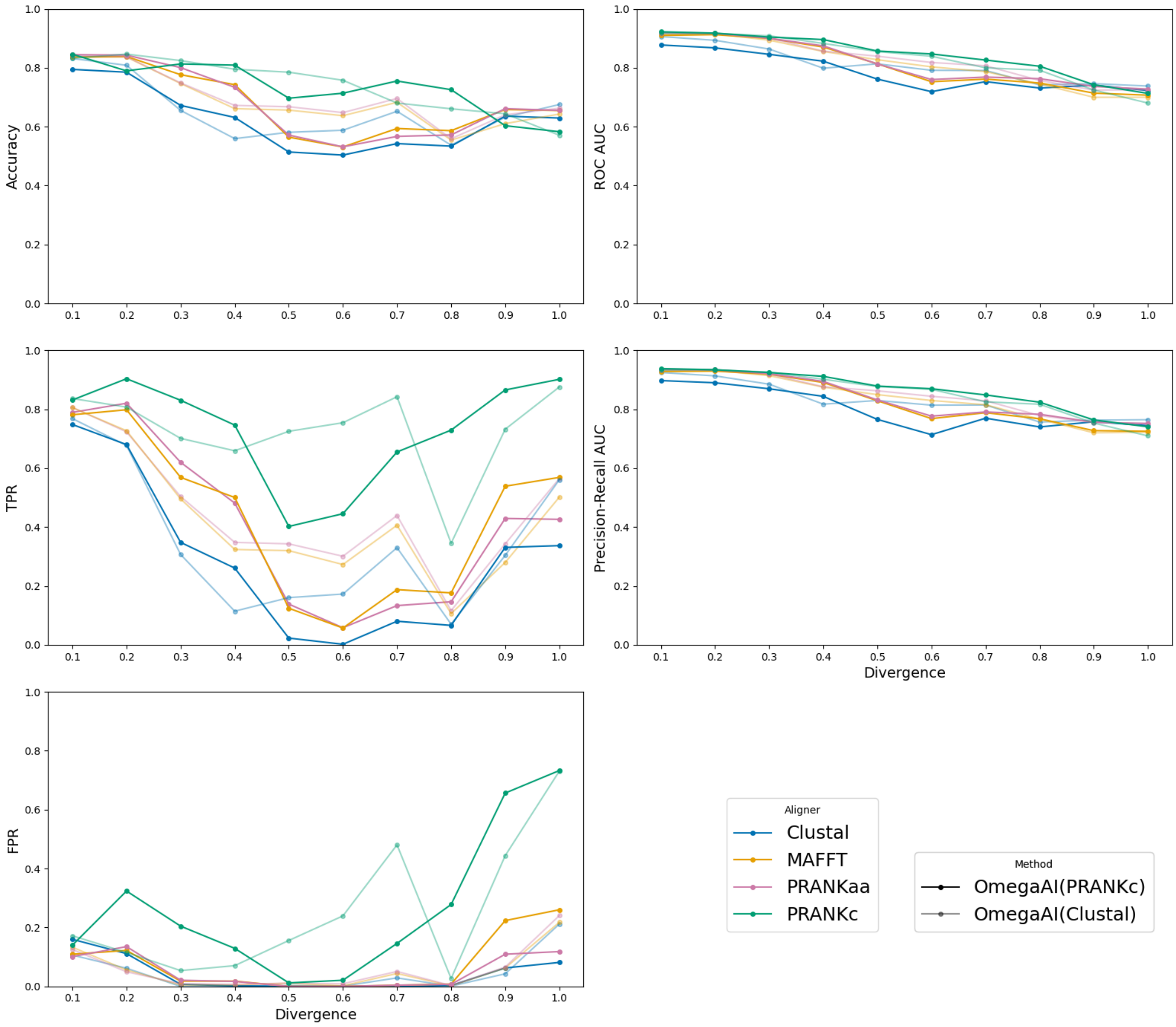


**Figure S 4. OmegaAI model trained on PRANKc alignments vs. OmegaAI model trained on Clustal alignments.** Various binary classifier performance metrics are presented to compare the two methods. In the first column of plots the accuracy, TPR and FPR are calculated under a threshold of 0.5 for both methods. The divergence axis refers to the scaling of branches of the 8-taxon symmetric tree (Supplementary Fig. 1) used for simulation, and a different OmegaAI model is trained for each divergence level and for each method. The baseline parameter for indel rate, 0.1, is used. The OmegaAI(Clustal) models are trained exclusively on Clustal alignments and shown in the semi-transparent lines, whereas OmegaAI(PRANKc) models are trained exclusively on PRANKc alignments and shown in the bold lines. The test data is aligned by the four different aligners and tested by both methods. The two sets of models perform comparably, indicating that there is little benefit from training on the more accurate PRANKc alignments, despite the considerable computational resource cost of running PRANK.

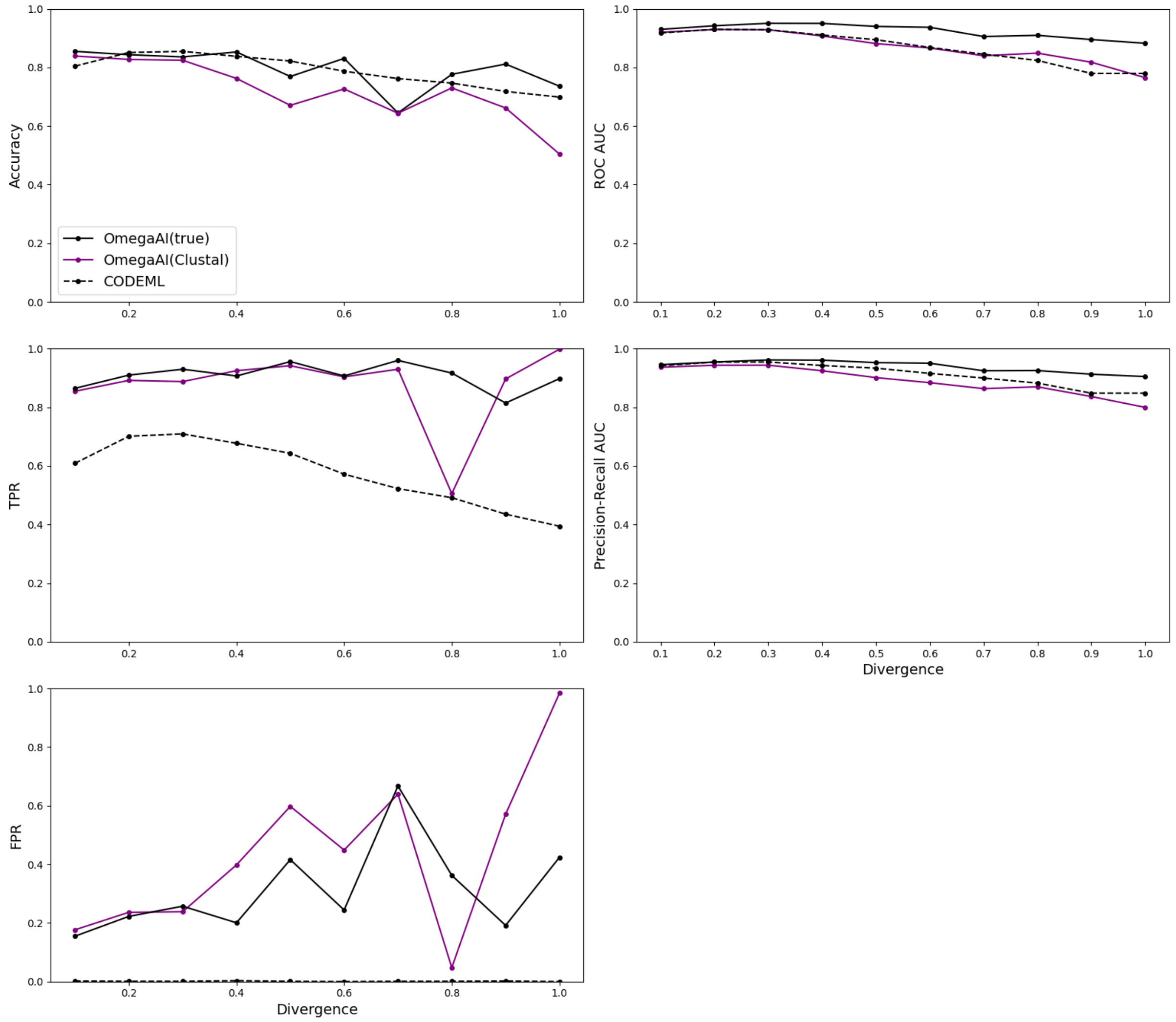


**Figure S 5. OmegaAI vs. CODEML — true alignments.** Various binary classifier performance metrics are presented to compare three methods: OmegaAI trained on true alignments or Clustal alignments, and CODEML. In the first column of plots the accuracy, TPR and FPR are calculated under a threshold of 0.5 for both AI methods, and a threshold of $p = 0.95$ for CODEML. The divergence axis refers to the scaling of branches of the 8-taxon symmetric tree (Supplementary Fig. 1) used for simulation, and a different OmegaAI model is trained for each divergence level and for each AI method. The baseline parameter for indel rate, 0.1, is used. All three methods are evaluated on the same set of 2,000 true alignments. In purple are the standard OmegaAI models trained on Clustal alignments. The bold black lines show results from OmegaAI models trained using true alignments; dashed lines are CODEML results. The three methods perform comparably, with the OmegaAI(true) models outperforming the standard OmegaAI models across metrics. The TPR and FPR suggest that the decision threshold could benefit from being lowered to reduce the FPR to come more in line with CODEML's conservative results.

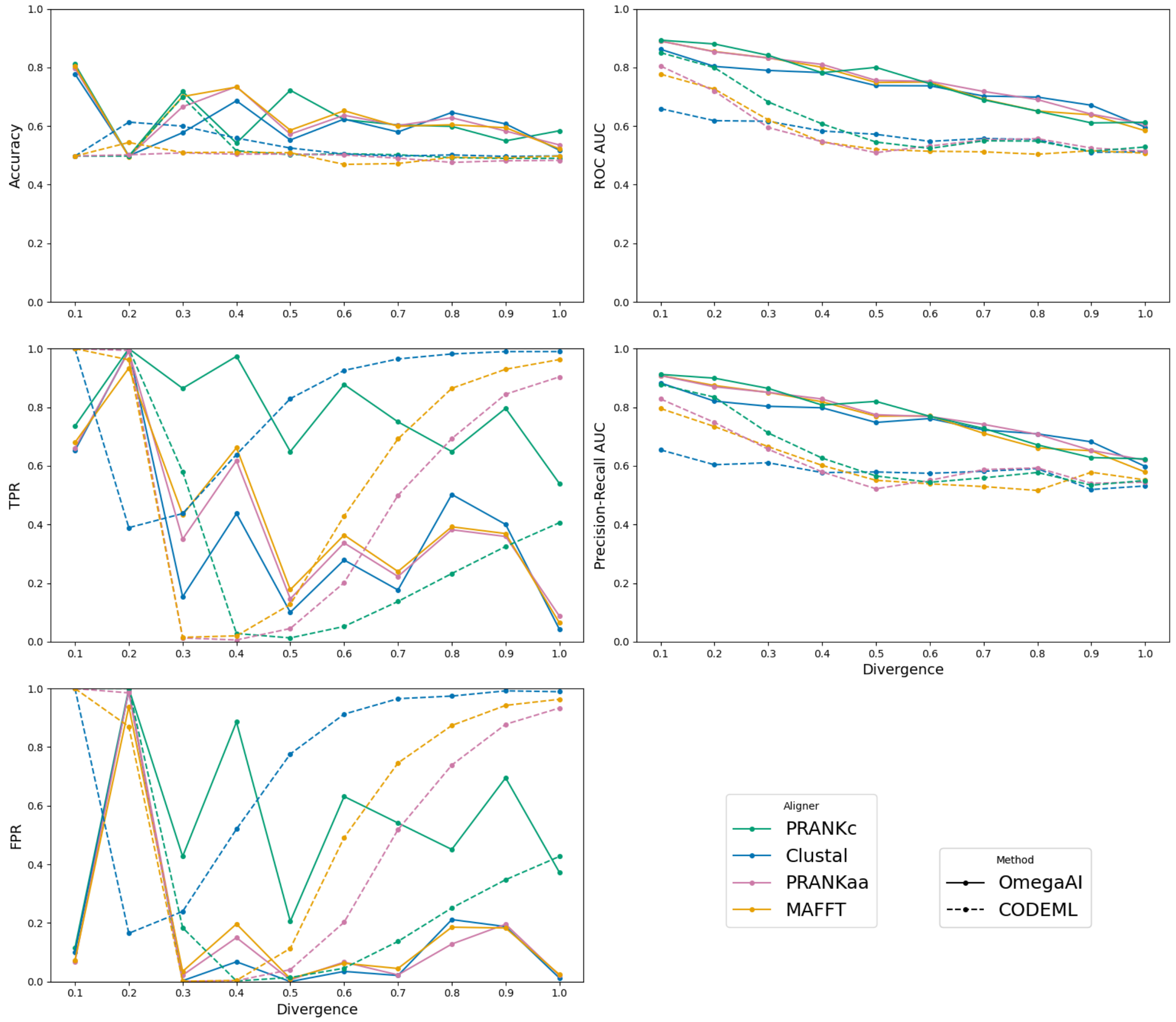


**Figure S 6. OmegaAI vs. CODEML — indel rate 0.2 across divergences.** Various binary classifier performance metrics are presented to compare the two methods. In the first column of plots the accuracy, TPR and FPR are calculated under a threshold of 0.5 for OmegaAI, and a threshold of $p = 0.95$ for CODEML. The divergence axis refers to the scaling of branches of the 8-taxon symmetric tree (Supplementary Fig. 1) used for simulation, and a different OmegaAI model is trained for each divergence level. A value of 0.2 is used for the indel rate. The OmegaAI models are trained exclusively on Clustal alignments. The test data is aligned by the four different aligners and tested by both methods. Increasing the indel rate from the baseline of 0.1 presents more variable trends and generally poorer performance from both methods, with OmegaAI still outperforming CODEML as shown by the ROC and precision-recall AUC values. We postulate that due to higher indel rates leading to lower quality alignments, it is harder for OmegaAI to distinguish between true and false positives. As a result, accuracy, TPR and FPR are more sensitive to the choice of threshold, leading to more variable fluctuations in TPR and FPR, whilst ROC and precision-recall AUC value trends are smoother.

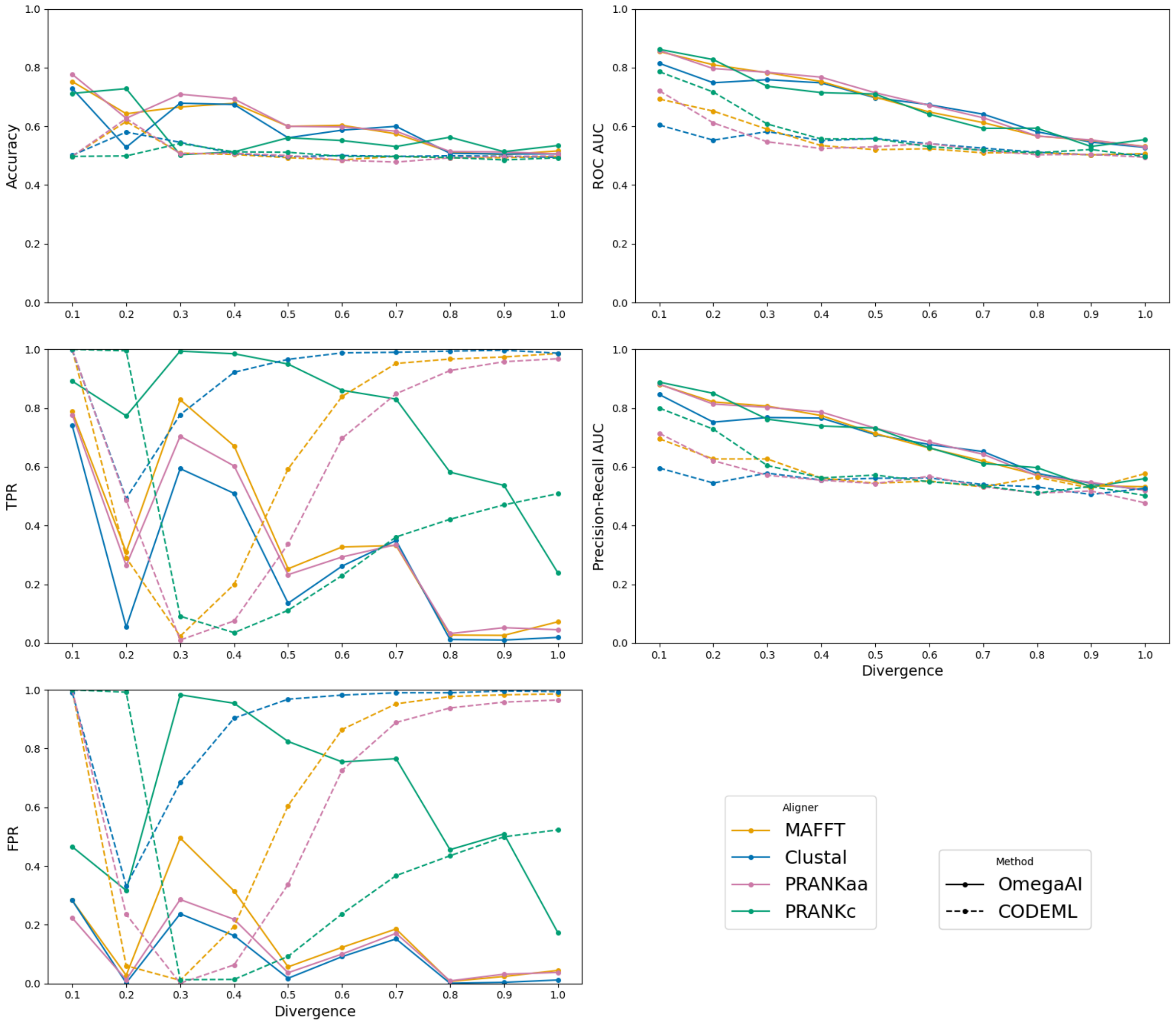


**Figure S 7. OmegaAI vs. CODEML — indel rate 0.3 across divergences.** Various binary classifier performance metrics are presented to compare the two methods. In the first column of plots the accuracy, TPR and FPR are calculated under a threshold of 0.5 for OmegaAI, and a threshold of $p = 0.95$ for CODEML. The divergence axis refers to the scaling of branches of the 8-taxa symmetric tree (Supplementary Fig. 1) used for simulation, and a model is trained for each divergence. A value of 0.3 is used for the indel rate. The OmegaAI models are trained exclusively on Clustal alignments. The test data is aligned by the four different aligners and tested by both methods. Increasing the indel rate from the baseline of 0.1 presents more variable trends and generally poorer performance from both methods, with OmegaAI still outperforming CODEML as shown by the ROC and precision-recall AUC values (explanation for this behaviour is as in Supplementary Fig. 6).

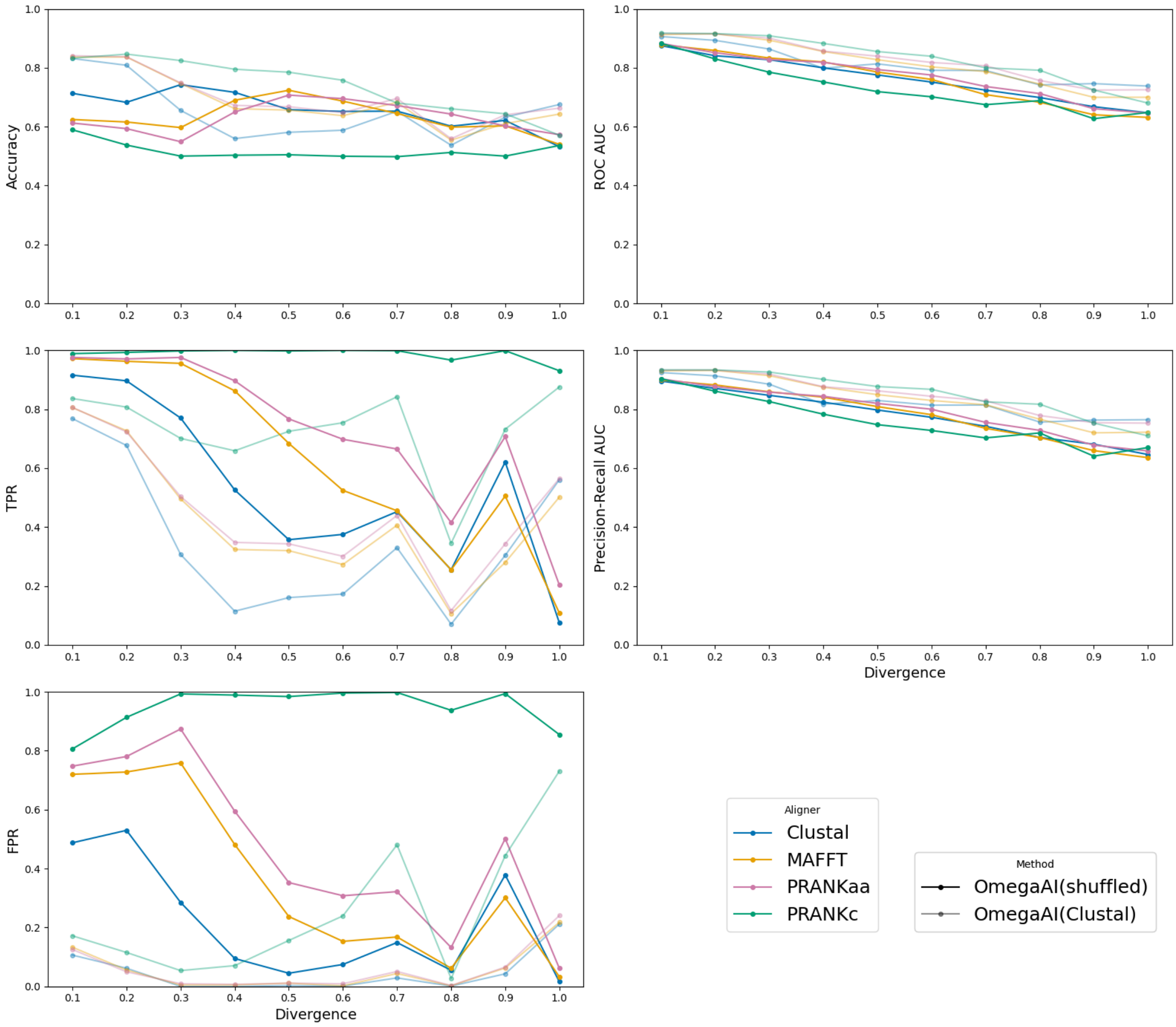


**Figure S 8. OmegaAI — shuffled vs. consistent MSA row ordering.** Various binary classifier performance metrics are presented to compare the two methods. Semi-transparent lines: results from the standard OmegaAI models that have been trained on Clustal alignments where row orderings are always consistent relative to the underlying simulation tree shown in Supplementary Fig. 1. Bold lines: results from OmegaAI models trained and tested on Clustal alignments where the row ordering has been randomised, meaning no information about the underlying tree is given to the models during training or testing. The divergence axis refers to the scaling of branches of the 8-taxa symmetric tree used for simulation. The baseline parameter for indel rate; 0.1, is used. These results show that the standard OmegaAI model leverages information about the tree in its learning and inference, and performance drops when it does not have access to this information.

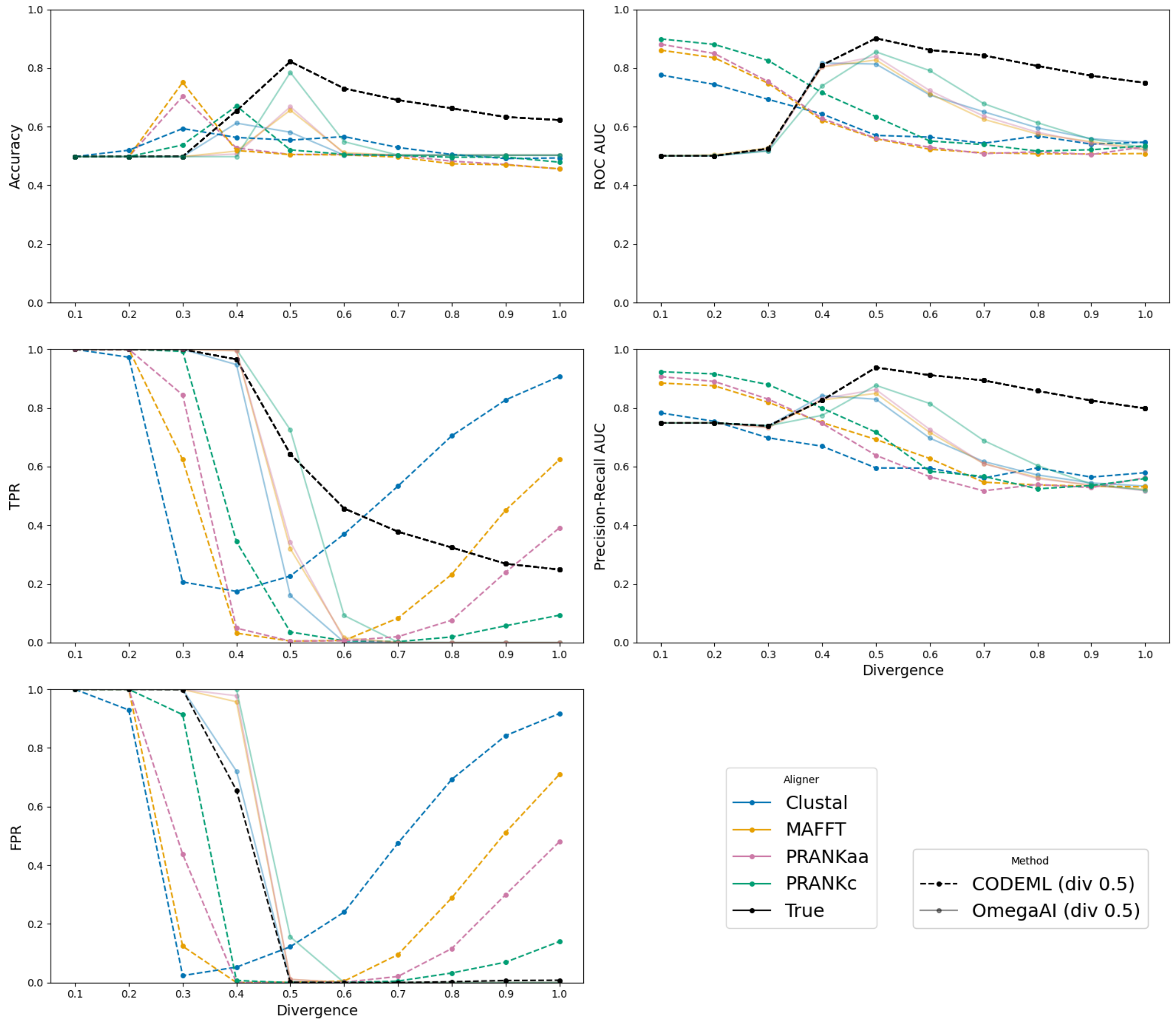


**Figure S 9. OmegaAI vs. CODEML — 0.5 divergence models.** Various binary classifier performance metrics are presented to compare the two methods. Semi-transparent lines show the OmegaAI model that has been trained on sequences simulated under the simulation tree with branches scaled to 0.5 substitutions per codon site per branch and aligned with Clustal. Dashed lines represent CODEML results, where CODEML has been forced to use the simulation tree with branches scaled to 0.5 during its free parameter value inference through maximum likelihood optimisation. Both OmegaAI and CODEML are tested with datasets across our divergence range, as indicated by the x-axis of each plot. Data at the divergence level of 0.5 is the only data for which the test data used has the same divergence level as is assumed by the methods. Colours represent the aligner used to align the test data. This analysis was undertaken to assess OmegaAI's ability to generalise to data that has evolved under different evolutionary rates compared to that which it was trained on. CODEML forced to use the 0.5 divergence tree is intended to be the analogous experiment in the maximum likelihood framework, for comparison. Both methods call positive selection for almost all data they are exposed to with divergence much less than 0.5. When tested using data with divergence greater than 0.5, OmegaAI tends to label almost all data with no positive selection. CODEML exhibits the opposite behaviour, where it overestimates positive selection and results in increasing TPR and FPR with increased divergence. The exception to this trend is CODEML applied to true alignments, where the trend looks more similar to that exhibited by OmegaAI, except CODEML is able to infer some true positives where OmegaAI struggles to do so.

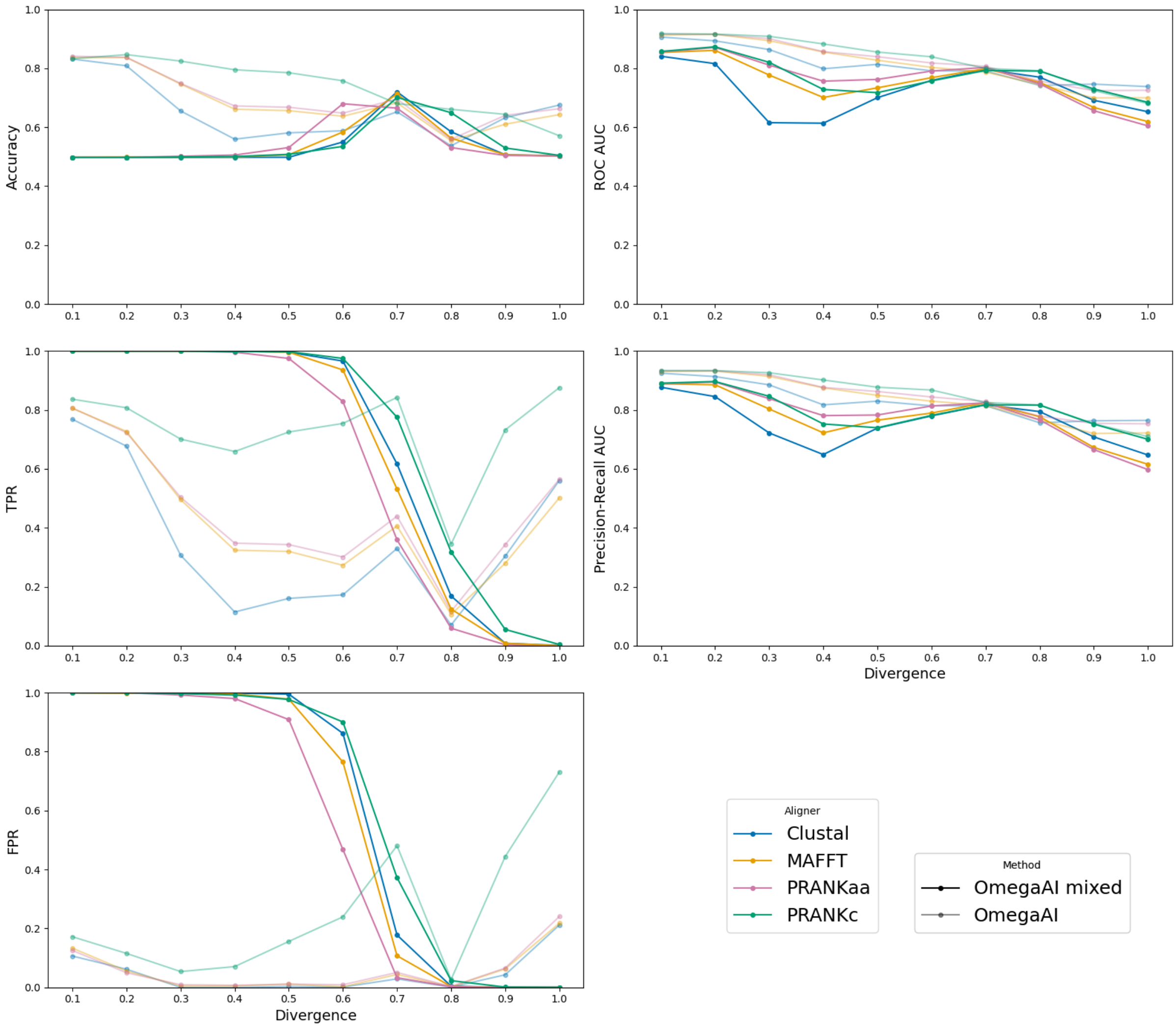


**Figure S 10. OmegaAI mixed model vs. individually trained OmegaAI models at each divergence.** Various binary classifier performance metrics are presented to compare the two methods. Semi-transparent lines show the results from individual OmegaAI models trained and tested for each divergence level in our divergence set, as seen in Fig. 4. Models are trained using Clustal alignments. Solid lines show an OmegaAI model that has been trained on an equal number of trees from each divergence level in the set, with 1,000,000 in total. This model is then also tested across our divergence set. The analysis shows that a single OmegaAI model trained on different divergences is not immediately generalisable to all divergence testing scenarios because there is a thresholding issue, as indicated by low accuracy but high AUC values.

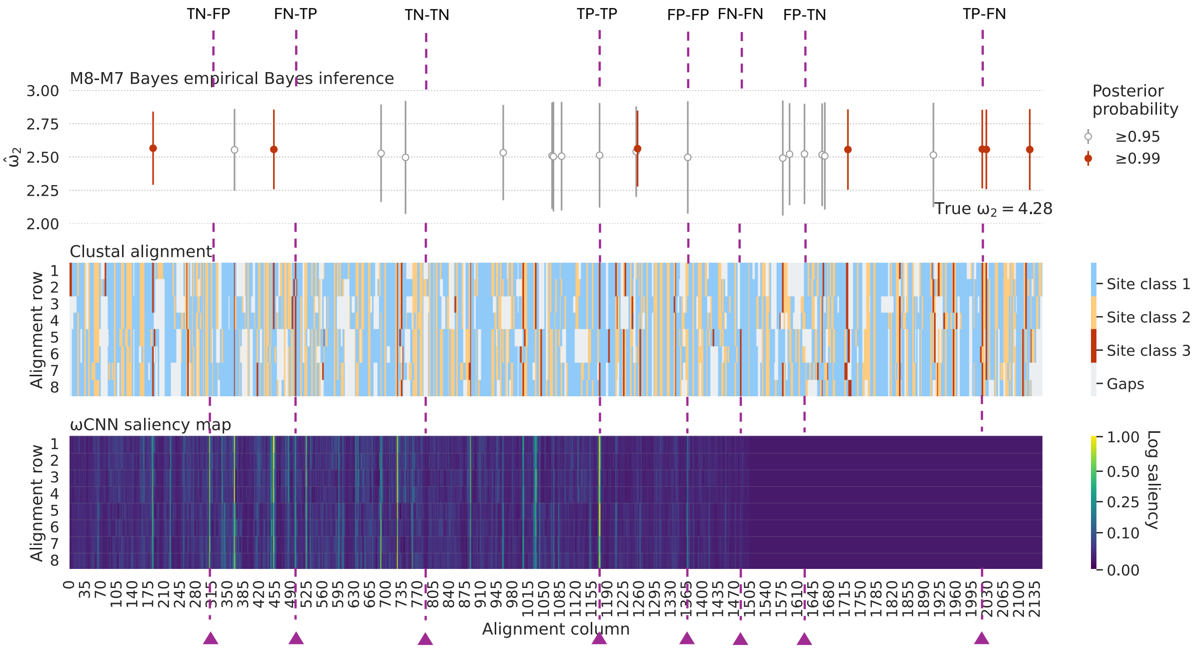


**Figure S 11. Comparing OmegaAI and CODEML inferences on sequences simulated under positive selection.** This plot and data is the same as seen in the main text Fig. 9, but with further annotation. Vertical lines label examples of each of the 8 possible combinations of true/false positive/negative combinations when comparing OmegaAI and CODEML sitewise inferences of positive selection. However, we note that the OmegaAI negative inferences at the far right of the MSA are likely due to zero-padding during training (see Discussion). **Top panel:** CODEML sitewise predictions of positive selection. Following significant results from both M1a/M2a and M7/M8 LRTs (as described in Methods), an empirical Bayes approach is then used to calculate the posterior probability that each site is from a particular site class. Inferences of sites belonging to site classes with $\omega > 1$ with $p \geqslant 0.95$ or $p \geqslant 0.99$ are shown by grey and red bars, respectively. **Middle panel:** Colour coded Clustal alignment. Blue, yellow and red represent the three site classes described in Methods, with red indicating sites where $\omega > 1$. Grey indicates gaps in the alignment. **Bottom panel:** OmegaAI saliency map, computed as outlined in Methods.

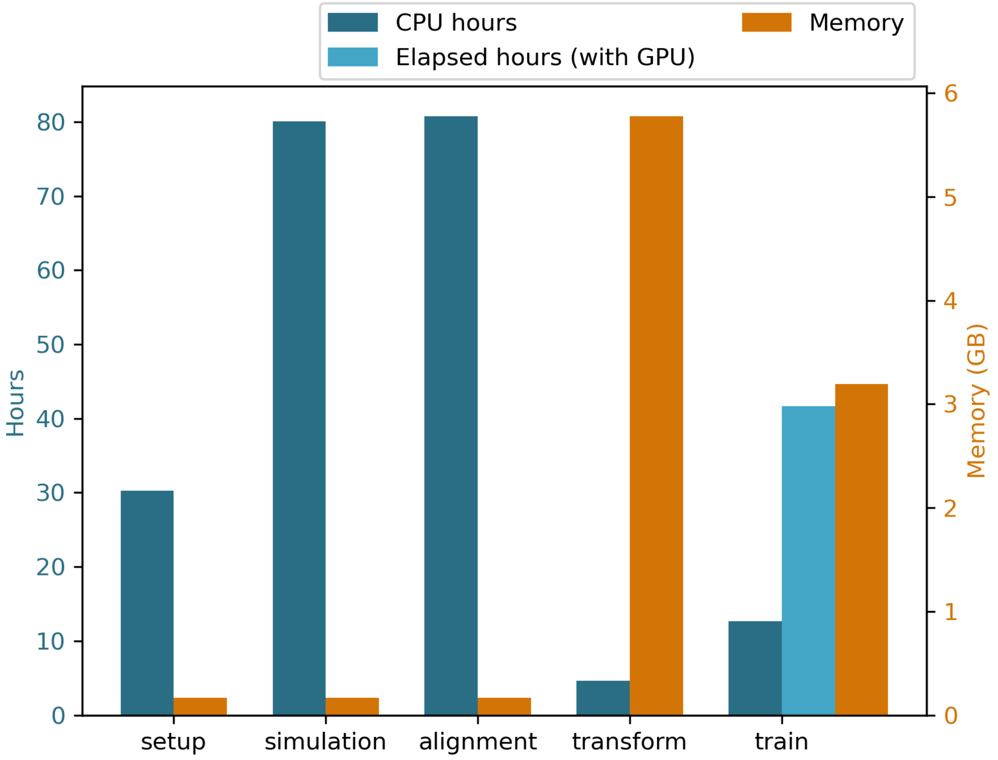


**Figure S 12. Benchmark resources for OmegaAI training workflow.** CPU hours reflects an estimate of the time each step would take if run sequentially on a single CPU. In practice, most steps permit some amount of parallelisation when resources allow. Memory describes the maximum Resident Set Size (maxRSS) — the peak amount of physical memory actively used by a process during execution of each step. The workflow for training an OmegaAI CNN is split into the stages setup, simulation, alignment, data transformation (transform) and train (see Fig. 2). "Setup" describes processes such as directory creation, list creation for data chunking and various I/O. For the baseline model, 1,000,000 alignments are simulated using INDELible (Fletcher and Yang, 2009) and re-aligned using Clustal Omega (Sievers, Wilm, et al., 2011). In our implmentations, both processes are typically allocated 2 CPUs and 512MB of memory. "Transform" describes the process whereby MSAs are converted to TFRecord format (typically allocated 1 CPU and 12GB of memory). Training the CNN involves a single CPU and a single GPU (we used NVIDIA Tesla V100 PCIe 32 GB). In this step, in addition to the total CPU time, we also include the elapsed hours to indicate the total time it takes to run the CNN training step (c. 41.6 hours), for most of which the GPU is primarily utilised (GPU utilisation c. 75%).

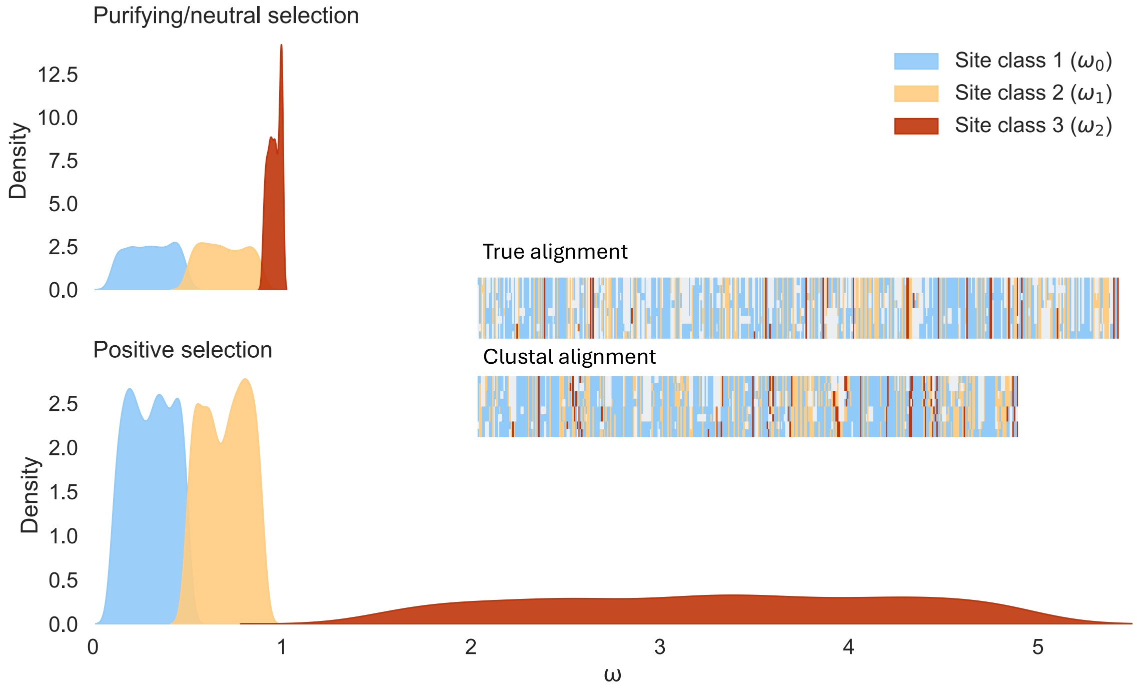


**Figure S 13. Distributions of $\omega$ values sampled for our "baseline" parameter simulations.** Kernel densities are shown to represent example distributions of $\omega$ values for the two simulation scenarios of purifying/neutral selection (top subplot) and positive selection (bottom subplot). These were created from empirically sampled $\omega$ values from the sampling distributions as defined in Methods. For purifying/neutral selection, $\omega$ values are always $\leqslant 1$. For positive selection, $\omega_2$ is always $\geqslant 1.5$ ($U(1.5, 5)$). An example is given of a colour-coded MSA from a true alignment and the same sequences re-aligned using Clustal. This illustrates how homologous codon sites become misaligned and the alignment becomes shorter due the mishandling of indels and over-alignment by Clustal.

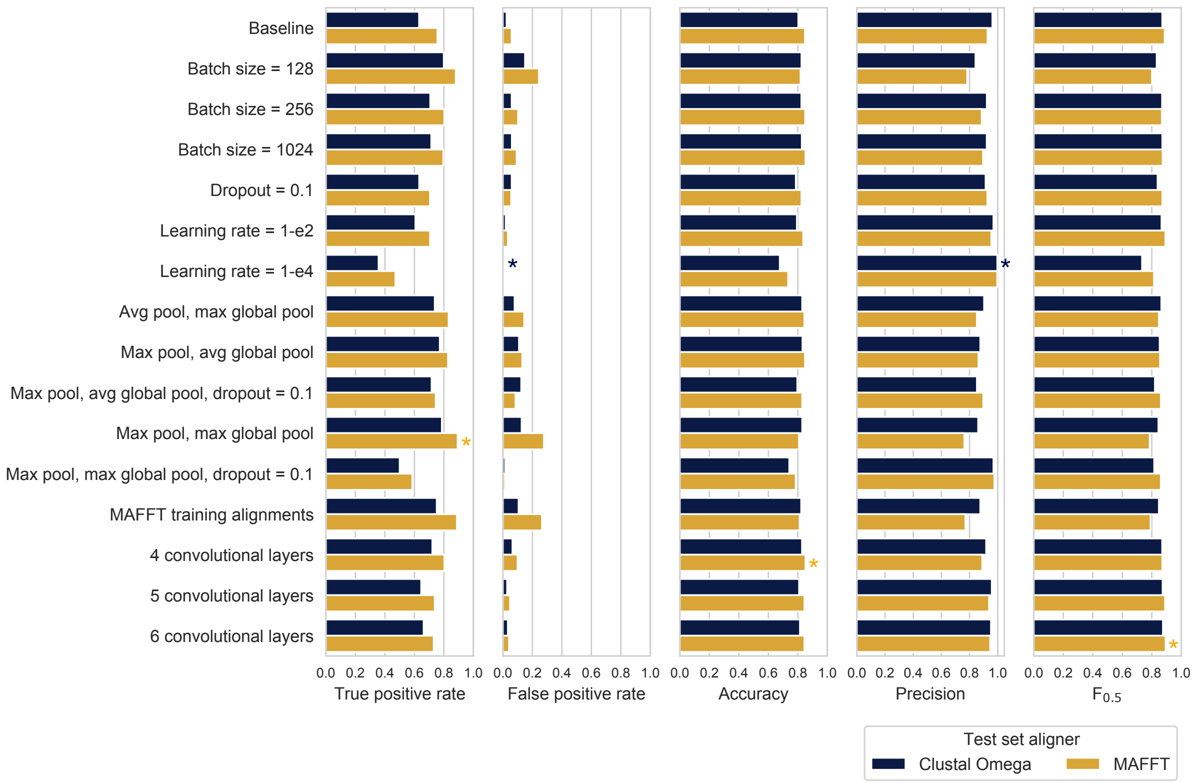


**Figure S 14. CNN performance with hyperparameter, training data, and architecture changes.** Starting with a "baseline" CNN architecture (top) and a "baseline" set of simulation parameters (see Methods), we trained the network for 50 epochs using 950,000 simulated alignments (re-aligned with Clustal Omega), and evaluated the performance of the trained network across a range of metrics (shown in separate subplots) by classifying a test set of 100,000 unseen Clustal Omega alignments, simulated using the same parameters as used for the "baseline" simulations. Then, for a selection of hyperparameters, including batch size, dropout probability, and learning rate, we re-trained the network and classified the same test set to compare performance. We also trained our network using MAFFT-aligned training data, different pooling operations, and with different numbers of convolutional layers. For each metric, an alignment colour-coded asterisk is shown beside the network/training data configuration which achieved the highest performance for that metric. Deviations from the "baseline" architecture and hyperparameter set yielded either inferior performance or only marginal improvements, depending on the metric. While networks with fewer convolutional layers occasionally showed slight improvements, these differences were minimal and likely influenced by factors such as random initialisation. Given that deeper architectures generally perform better, we retained the baseline architecture with seven convolutional layers as the standard OmegaAI model for this study, except in cases where alternative training data are explicitly specified.

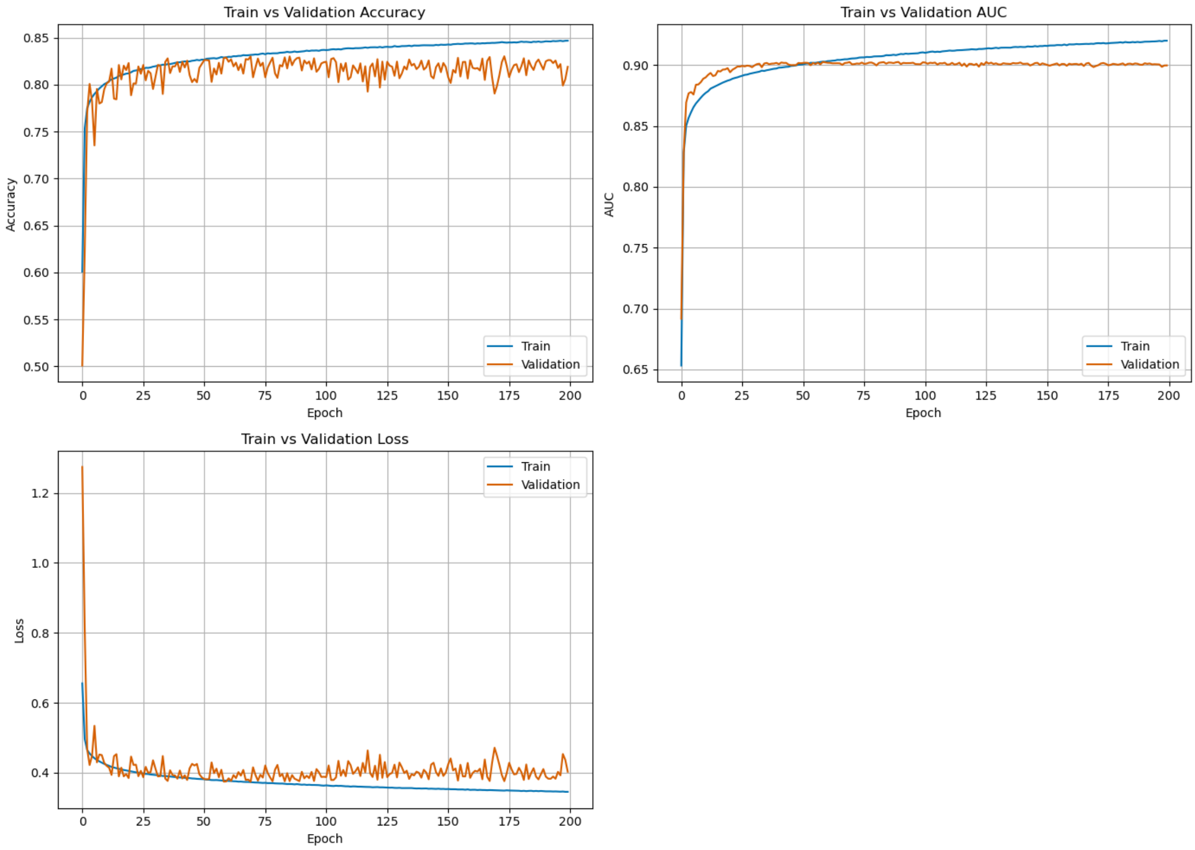


**Figure S 15. Training metrics over 200 epochs.** Accuracy, AUC and loss shown for both training (950,000 MSAs) and validation (50,000 MSAs) sets when training a baseline OmegaAI model over 200 epochs. Based on these analyses, the OmegaAI models in this study were trained for 50 epochs. This early stopping point prevents overfitting and avoids expending additional resources for negligible performance gains.

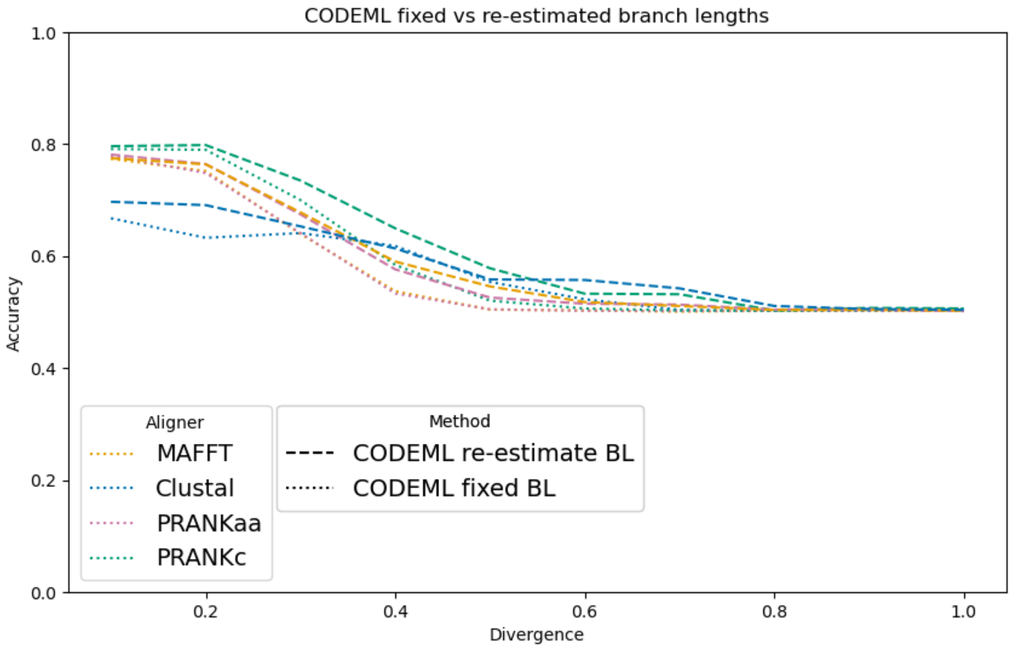


**Figure S 16. Comparing CODEML performance with fixed vs. re-estimated branch lengths.** CODEML was evaluated on sequences simulated under baseline conditions but with increasing divergence, for two scenarios, on four aligners. In both scenarios, CODEML is given the simulation topology of Supplementary Fig. 1 as the initial guide tree. In one scenario, during maximum likelihood estimation of parameters, CODEML is allowed to re-estimate branch lengths (results shown by dashed lines). In the other scenario the branch lengths are fixed (results shown by dotted lines). Across aligners and divergences, CODEML achieves higher accuracy when it is allowed to re-estimate branch lengths (as is common practice): this is the method presented throughout this work.
